# Ionic Liquids as Tools to Incorporate Pharmaceutical Ingredients into Biopolymer-Based Drug Delivery Systems

**DOI:** 10.3390/ph16020272

**Published:** 2023-02-11

**Authors:** Paula Berton, Julia L. Shamshina

**Affiliations:** 1 Chemical and Petroleum Engineering Department, University of Calgary, Calgary, AB T2N 1N4, Canada; 2 Fiber and Biopolymer Research Institute, Department of Plant and Soil Science, Texas Tech University, Lubbock, TX 79409, USA

**Keywords:** active pharmaceutical ingredients, biopolymers, ionic liquids

## Abstract

This mini-review focuses on the various roles that ionic liquids (ILs) play in the development and applications of biopolymer-based drug delivery systems (DDSs). Biopolymers are particularly attractive as drug delivery matrices due to their biocompatibility, low immunogenicity, biodegradability, and strength, whereas ILs can assist the formation of drug delivery systems. In this work, we showcase the different strategies that were explored using ILs in biopolymer-based DDSs, including impregnation of active pharmaceutical ingredients (APIs)-ILs into biopolymeric materials, employment of the ILs to simplify the process of making the biopolymer-based DDSs, and using the ILs either as dopants or as anchoring agents.

## 1. Introduction

Ionic liquids (ILs, salts with melting points below 100 °C), are widely used as “tailored” systems in broad areas of chemistry and chemical engineering, including synthesis [1,2,3,4,5], catalysis [6,7], and environmental remediations [8]. In the case of pharmaceutical applications [9], active pharmaceutical ingredients (APIs) are made into ILs (API-ILs) to produce liquid and more bioavailable forms of APIs [10,11], or ILs are used for API delivery [12,13]. The concept of API-ILs was first published in 2007 [9,14] and was illustrated with hundreds of examples afterwards. As of today (January 2023), SciFinder shows 98 patents, 2177 research papers, and 289 review papers using the search term “API-ILs”. It was proposed that API-ILs could help overcoming critical issues of the solid-state APIs in the pharmaceutical industry and regulatory agencies—such as polymorphism and/or poor solubility [15,16]. These known yet unpredictable problems present difficulties in handling, formulating, and testing solid-state APIs. Further, APIs can crystallize in the form of solvates and hydrates which can also have polymorphs with different solubilities [17,18] and bioavailabilities [19,20]. The classic example is Ganciclovir, an antiviral guanine nucleoside that has two polymorphic forms and two hydrates [21,22] significantly less water-soluble than its originally marketed form [23]. Another example is the accidental discovery of polymorph II of Ritonavir which resulted in its market withdrawal [24]. When API(s) become part of the IL, the API-IL itself is suitable for the administration of the API without requirement of any additional components in the formulation. A few exhaustive reviews available on the topic of API-ILs will help the reader familiarize themselves with the progress made using this approach, mainly focused on improvements in API solubility and bioavailability [25,26,27]. 

In addition, ILs have attracted interest as materials for drug formulations and delivery due to their inherent tunable physicochemical and biological properties [28]. ILs have been used as stabilization media, solvents, or cosolvents for drugs, affecting both their aqueous solubility and pharmacokinetics. ILs have proved to be effective in the dissolution of poorly water-soluble APIs: As an example, rutin, a flavonoid with anti-inflammatory, anticancer, antidiabetic, and antibacterial activity, exhibited much higher (6-fold) solubility when dissolved in choline 2-hydroxyethyl-trimethylammonium-L-phenylalanine ([Cho][Phe]) and choline 2-hydroxyethyl-trimethylammonium-L-glutamate ([Cho][Glu]) [29]. 

Many APIs are supplied using drug delivery systems (DDSs) in the form of formulations (e.g., emulsions) or delivery matrices so that the drug can safely travel within the human body [30]. ILs have been able to successfully enhance the skin permeation of several drugs. Recently, biocompatible ILs mainly focused on choline-based ILs with enhanced toxicological and ecological features received a lot of attention [31,32]. ILs are also able to form microemulsions for drug delivery, with imidazolium ILs of low cytotoxicity profiles being widely used for the purpose [33,34,35,36]. In addition to microemulsions, other formulation strategies were employed such as ionogels, thermo- or pH- responsive gels, surfactant-containing formulations, etc. [37,38,39].

An appropriate drug carrier should have high stability, a reasonable lifetime in the systemic circulation, allow for homogeneous distribution of a drug [40], and release the drug at an anticipated rate. API-ILs have been loaded into mesoporous silica or silica-based ionic gels for controlled drug release [37,41]. Polymeric or mesoporous silica excipients improve the stabilization of the drug. Particularly, polymers allow for molecular dispersion with the drug limiting its mobility and inhibiting crystal growth and nucleation [42]. However, the design of such delivery matrices is nowadays focused on natural systems (e.g., biopolymers) [43,44]. The major advantages of using biopolymers as DDSs are their biocompatibility, bioactivity [45], strength [46,47], and biodegradability [48]. For example, cellulose is a relatively inert, mechanically strong, and biocompatible polymer [49,50,51]. It possesses a supramolecular 3D ultrafine highly porous fiber network which is suitable for drug dispersion, selectively permeable, and highly water-absorbent [52]. Chitin [53], a biopolymer structurally similar to cellulose with an acetamide group on the C-2 atom, is an attractive material as DDS matrix [54] due to its remarkable strength. Chitin is non-toxic, biodegradable, and non-allergenic [55,56]. Chitin derivative, chitosan, prepared by deacetylation of chitin, displays most of the material characteristics similar to those of chitin, although it has a lower tensile strength and modulus than chitin. Chitosan is inherently antibacterial.

Since 2002, the ILs have been used for biopolymer processing, when solubilization of cellulose in 1-butyl-3-methylimidazolium chloride ([C_4_mim]Cl) was discovered. Such solubilization was found to be a result of the polymer’s hydrogen bonding network disruption [57,58]. Because of such disruption [59], the dissolution is believed to be a physical, rather than a chemical, process [60,61], thus, upon dissolution, the polymers are not degraded and possess high molecular weight (MW). This discovery generated intense worldwide academic and industrial interest and, as the field progressed, it was found that ILs are suitable for the dissolution of chitin, chitosan, silk keratin, starch, lignin, and so on [62,63,64,65]. ILs have been exploited to prepare matrices, scaffolds, and composite materials in a form of membranes, microbeads, nanomats, etc. through solution processing techniques [66,67] such as spin-, dip-, or spray-coating, casting, molding, 3D printing, jet wet- and dry-spinning, and electrospinning [68,69,70,71,72].

The most exciting scientific endeavors involve the merging of two or more separate research areas into a single research project, and the ILs often facilitate such fusions. The thought of combining both areas—biopolymeric materials and API—through anchoring or embedding API into biopolymer-based products using ILs is the subject of this mini-review. Because biopolymers are suitable for a large variety of biopolymer-based functional materials, all of these can be used as drug-delivery vehicles. There are multiple facets of the process that can be facilitated and even simplified, with the use of ILs. Thus, ILs have been shown to be suitable for dissolution, co-dissolution and/or co-dispersion of bio- and synthetic polymer(s), to prepare composite matrices [73]; ILs are used for co-dissolution and/or co-dispersion of biopolymers with pharmaceutical additives [74]; biopolymers can be surface-functionalized with APIs through either covalent (in case of chitin, cellulose) or ionic (in case of chitosan) derivatization [75]. Materials’ properties could be tailored based on the identity and characteristics of constituent (bio)polymer(s), the ratio between them, kinetics of network formation, and types of crosslinking, to name a few factors. In this mini-review, we will report strategies combining the approaches above, highlighting the versatility of ILs.

## 2. Drug Delivery Systems Using Biopolymers as Delivery Matrices and ILs as Formulation Components

### 2.1. Incorporation of API-ILs into Biopolymeric Materials

Biopolymers such as chitosan and bacterial cellulose (BactCel) allow their manipulation into materials without the need for their dissolution in harsh chemicals or ILs. Bacterial nanocellulose (BactCel) membranes were loaded with phenolic acid-based ILs [76]. The ionic compounds, with improved solubility and bioavailability, were prepared by combining the choline cation with anions derived from ellagic, gallic, and caffeic acids to form choline ellagate ([Cho]_2_[Ell]), choline gallate ([Cho][Gal]), and choline caffeate ([Cho][Caf]), respectively. BactCel membranes were then soaked in 1 mL of aqueous solutions containing 10 mg/mL [Chol][Caf], [Chol]_2_[Ell], or [Chol][Gal] and dried under a nitrogen atmosphere. The obtained BactCel-ILs membranes showed a controlled ILs dissolution rate in the wet state and high antioxidant activity. In vitro assays performed with Raw 264.7 macrophages and HaCaT keratinocytes revealed that these BactCel-ILs membranes are non-cytotoxic and exhibit anti-inflammatory activity. Diffusion studies with Hanson vertical diffusion cells showed a prolonged release profile of the ILs from the BactCel membranes, demonstrating their potential for topical applications (e.g., post-surgery treatments and burn and wound healing) [76]. This same approach, i.e., synthesizing API-ILs and soaking BactCel membranes in these API-ILs, was used to load API-ILs made of NSAIDs, such as choline ibuprofenate, choline ketoprofenate, and choline naproxenate into BactCel membranes [77]. The rehydration ability of IL-incorporated membranes was found to be 18 to 26 times higher than BactCel, exhibiting superior performance in the absorption of exudates. Release tests demonstrated a faster and complete release of the IL-based drugs when compared with the release of non-ionized NSAIDs. Finally, it was shown that BactCel is not cytotoxic nor proinflammatory, whereas the cytotoxicity and anti-inflammatory properties of the IL-incorporated BactCel membranes were similar to those of the original NSAIDs or ILs, reinforcing their suitability as materials for topical drug release applications, such as delivery patches [77].

A novel solution blend approach was used to prepare hybrid gels consisting of viscoelastic gels of the API-IL 1-dodecyl-1-methylpiperidinium acetylsalicylate ([C_12_mpip][AcSa]) in sodium salicylate aqueous media blended with the biopolymers agar or chitosan with low (L) and medium (M) Mw [78]. The mixtures formed physical networks due to the polymer chain entanglement. The amount of drug released from the viscoelastic micellar solutions without biopolymers was high, while the amount of the drug released from hybrid systems followed the order: Agar > chitosan (L) > chitosan (M), confirming that chitosan of high Mw forms more compact collar flexible network which traps the drug molecules and makes its release slower. Similarly, the slight decrease in the amount of drug released with the increase in the concentration of biopolymer was explained by an increase in the density of networks, slowing down the release of the drug [78].

### 2.2. ILs as Preparation Media

The most often reported strategy for making biopolymer-based materials as drug carriers uses a two-step approach, where ILs or DESs are utilized to dissolve and/or modify the biopolymer to prepare the material, followed by the drug loading into the already-formed material. On the other hand, reports where ILs are used to both solubilize the biopolymer and, at the same time load the drug, are limited. 

Jet spinning, used to make nanofibers from an IL solution, could facilitate the formation of nanomaterials from the biopolymer solution using high-speed airflow as the driving force. Upon extrusion of the spinning solution through the spinneret, a polymer jet is formed under the action of high-speed airflow. Jet spinning has a higher spinning efficiency than electrospinning and, due to the driving effect of high-speed airflow, the resulting fiber has a finer diameter. The method was used for the jet spinning of cellulose. Besides cellulose as the main biopolymer, the biopolymer-IL ([C_4_mim]Cl or [C_4_mim]Br) spinning dope could contain organic solvents (e.g., ethanol), a second biopolymer (e.g., chitosan), and an active (e.g., honeysuckle extract), among other components [79]. After jet spinning, the obtained nanofibers were reported to have excellent hemostasis and wound healing properties, and because of being made of cellulose, the fibers could exist in the human body for a long time, achieving a sustained release effect.

**Figure 1:**
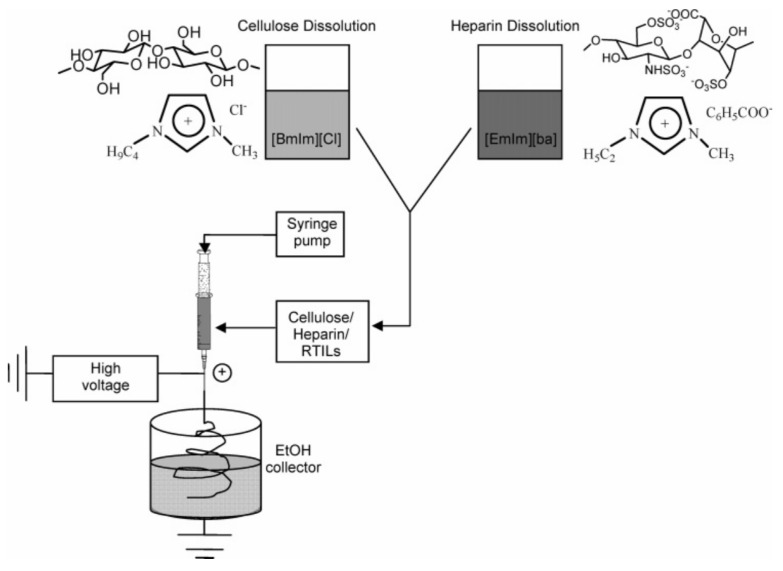
Schematic representation of the electrospinning process used to make heparin-cellulose fibers from the IL solutions. Reprinted with permission from [80]. Copyright 2006 American Chemical Society.

Heparin, a linear, polydisperse, anionic polysaccharide that plays a vital role in regulating many biological activities, has been included in cellulose-based materials [75,76,77,78,79,80,81,82]. This drug is the most widely used anticoagulant and has also been extensively investigated to prepare various blood-contacting polymer devices with good blood compatibility. Due to its limited solubility in organic solvents (soluble only in a few solvents including dimethylformamide, dimethyl sulfoxide, and formamide), heparin was turned from sodium heparin to imidazolium heparin and dissolved in 1-ethyl-3-methylimidazolium benzoate ([C_2_mim][C_6_H_5_COO]) (Figure 1). Separately, cellulose was dissolved in the IL [C_4_mim]Cl. Both the IL solutions, i.e., 10 wt% cellulose in [C_4_mim]Cl and 2 wt% heparin in [C_2_mim][C_6_H_5_COO], were mixed to get a clear cellulose-heparin solution, which was electrospun [80]. The performance of the cellulose-heparin composite fibers was then tested by measuring the clotting kinetics of human whole blood exposed to these fibers using thromboelastography (TEG). The presence of heparin in the fibers acted as an anticoagulant, slowing clot formation without altering the final amount of clot formed. Notably, heparin maintained its bioactivity even with exposure to high voltage during electrospinning (10–20 kV) [80]. 

Due to its anticoagulant property, the use of heparin in biopolymeric composite materials is also reported as a strategy to increase the biocompatibility of such materials. The reason is that cellulose itself is biocompatible but not completely blood compatible, while heparin would help avoid blood clotting. For example, this same approach was used to make heparin–cellulose–charcoal composites, to enhance the biocompatibility and blood compatibility of activated charcoal beads while decreasing the size of their active pores [81]. The 1 wt% cellulose and 0.5 wt% heparin in IL ([C_4_mim]Cl + [C_2_mim][C_6_H_5_COO]) solution was prepared as described above. After that uncoated activated charcoal beads (100 mg, prepared from resin pyrolysis) were added to the heparin–cellulose solution. The resulting suspension was loaded into syringes and introduced dropwise into excess ethanol to selectively remove the IL. This coating decreased the active pore size of the activated charcoal, thus diminishing its rate of protein adsorption, without decreasing the effective removal of free-diluted and protein-bound small drug molecules. These composites were proposed to remove small, hydrophobic protein-bound drug molecules rapidly and safely from the digestive system or from the blood of overdose patients in an extracorporeal circuit [81].

**Figure 2:**
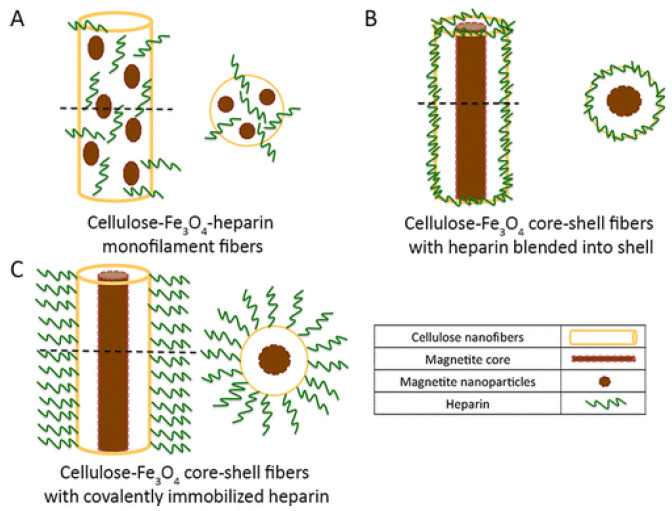
Structure of nanofibers. (**A**) Cellulose-Fe_3_O_4_-heparin monofilament fibers with entrapped Fe_3_O_4_ NPs and heparin. (**B**) Cellulose-Fe_3_O_4_ core−shell nanofiber with internal Fe_3_O_4_ NP core and cellulose shell blended with heparin. (**C**) Cellulose-Fe_3_O_4_ core−shell nanofiber with internal Fe_3_O_4_ NP core and cellulose shell with covalently immobilized heparin. Reprinted with permission from [82]. Copyright 2016 American Chemical Society.

Using IL-based processes allows versatility in the material preparation practices. For example, by incorporating superparamagnetic iron oxide nanoparticles (Fe_3_O_4_ SPIONs) into a cellulose matrix modified with heparin [82]. In this case, cellulose pulp was dissolved in 1-ethyl-3-methylimidazolium acetate ([C_2_mim][OAc]) or cellulose−Fe_3_O_4_−heparin solutions could be prepared by first dispersing Fe_3_O_4_ NPs in [C_2_mim][OAc] and then adding cellulose pulp and sodium heparin into the homogeneous NP solution. The heparin-coated cellulose nanofibers were synthesized by wet−wet electrospinning with Fe_3_O_4_ SPIONs incorporated to allow for the movement of the nanofibers produced through the application of an external magnetic field. Three synthetic routes were used to prepare three distinct types of nanocomposite fibers (Figure 2): cellulose−Fe_3_O_4_−heparin monofilament fibers, cellulose−Fe_3_O_4_−heparin core−shell fibers with heparin covalently immobilized on the fiber surface, and cellulose−Fe_3_O_4_ core−shell fibers with heparin physically immobilized on the fiber surface. Using this approach, cellulose nanofibers well tolerated in biological systems have been made suitable for direct blood contact and can be externally manipulated through a non-invasive application of a magnetic field [82]. 

### 2.3. System Based on Biopolymeric Matrices Doped with IL as Adsorption/Desorption Matrices for APIs

A frequent observation from the reviewed literature is that in the absence of an IL as a modifier, the drug release is governed by ionic and hydrogen bond interactions between the biopolymer and the API. In the case of chitosan, with pKa 6.3–6.5, the interactions, i.e., the extent of ionic and hydrogen bonding or only hydrogen bonding, and hence the drug release will be conditioned by the pH of the release media and the pKa of the API (if any) [64,65]. On the other hand, when one of the constituents of DDS is an IL, the interactions between IL and an API can be tuned to govern the drug release. One of the examples of DDS composed of both the polymeric matrix and an IL was developed for the delivery of an ionic API, sodium phosphate dexamethasone (DXA) [83]. For this study, chitosan was employed as a polymer, and choline chloride ([Cho]Cl) and choline dihydrogen phosphate ([Cho][H_2_PO_4_]) ILs were embedded into chitosan films. ILs were chosen for their ability to interact with both the chitosan matrix (to create a stable DDS) and the drug (to affect the release profiles of DXA). To prepare DDS, low Mw chitosan (Mw 145 kDa) was dissolved in an acidic solution which was then loaded with various amounts of the ILs. After that, DXA was added to [Cho]Cl- and [Cho][H_2_PO_4_]-containing chitosan solutions, stirred at room temperature, and solutions were cast into films. After solvent evaporation, the films were washed and dried. Pure chitosan films and films with embedded ILs and no drug were studied as controls.

DXA release kinetic studies from film samples were only studied for films loaded with [Cho][H_2_PO_4_] and conducted in three buffers of different pH (4, 7, and 10) as the release media, at 37 °C (body temperature). It was found that the addition of [Cho][H_2_PO_4_] helped slow down DXA release from the chitosan matrix, independently of the pH of the release medium. These differences were around 17% at pH 4, 10% at pH 7, and 28% at pH 10. These results demonstrate that the presence of the IL and its amount is an additional variable to be tuned for the sustained delivery of drugs. Although the mechanism was not entirely established, it could be hypothesized that ILs acted as solubilizers and stabilizers of DXA, and strong [Cho][H_2_PO_4_]–DXA interactions resulted in slowing DXA delivery from chitosan-based materials. In addition, the presence of the IL affected the film’s properties, such as strength, elastic moduli, and water vapor absorption and water vapor transmission rates (WVTR). Besides, the incorporation of the IL significantly improved the conductivity of the film and the sensitivity to electric stimulation under an electric field. The increased conductivity proposes the potential use of these films as stimuli-responsive drug delivery systems for applications such as iontophoresis. 

A similar study investigated microparticles (MPs) of chitosan cross-linked with tripolyphosphate and impregnated with ILs as a DDS for insulin [84]. Four different ILs were used for DDSs preparation: [Cho]Cl and imidazolium ILs, namely 1-butyl-3-methylimidazolium hydrogen sulfate ([C_4_mim][HSO_4_]), 1-butyl-3-methylimidazolium acetate ([C_4_mim][OAc]), and [C_4_mim][Cl]. To prepare chitosan tripolyphosphate MPs, each IL was mixed with a solution of chitosan in acetic acid and stirred for 1 h for homogeneity. After that, the pre-made insulin solution was mixed with the solution of chitosan in acetic acid and tripolyphosphate was added to initiate cross-linking, after which the mixture was stirred for 1 h. The prepared MPs were collected by centrifugation, washed repeatedly with DI water, and finally freeze-dried. It was found that MPs prepared with imidazolium ILs were highly swelled and exhibited low stability, hence, the insulin release kinetics was only studied for DDS containing [Cho]Cl, in 0.10 N HCl (pH = 1.2) and PBS buffer (pH = 7.4) at 37 °C (body temperature) for 8 h. The DDS that contained 0.10% [Cho]Cl resulted in a sustained insulin release rate with no burst release at pH = 1.20 and at pH 7.4. However, the insulin release after 8 h was lower at pH 1.2 than at pH 7.4 (49.3 vs. 78.2%, respectively), with the lowest rate constant (k) in the acidic medium, which was attributed to a release dependence mainly on the relaxation of the polymer structure as compared with the diffusion mechanism. The dominance of the release mechanism changed from diffusion to erosion (polymer relaxation) over time.

### 2.4. Systems Based on Anchoring ILs onto Polymeric Surfaces as Adsorption/Desorption Matrices for Ionic APIs

**Scheme 1:**
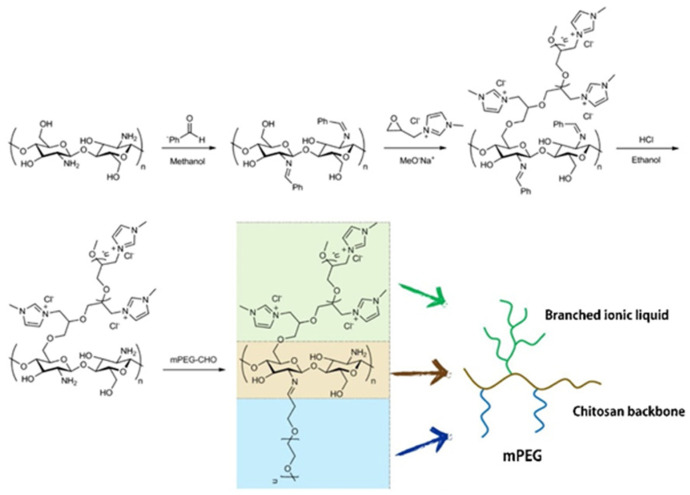
Schematic representation of preparation of multi-branched IL-based chitosan grafted m-PEG to coat the surface of Fe_3_O_4_ NPs for simultaneous delivery of Doxorubicin (DOX) and Methotrexate (MTX) to MCF7 breast cancer cells [85]. Reproduced with permission from [85].

The first system that illustrated anchoring ILs onto a polymeric surface was based on multi-branched IL-based chitosan grafted m-PEG to coat the surface of Fe_3_O_4_ NPs for simultaneous delivery of Doxorubicin (DOX) and Methotrexate (MTX) to MCF7 breast cancer cells [85]. To create DDS, amine functional groups of chitosan were first protected with benzaldehyde (to favor O-nucleophilic addition at C-6 carbon atom), isolated and re-dissolved in DMF. Then, 3-methyl-1-(oxiran-2-ylmethyl)-1H-imidazolium chloride was added to the reaction mixture and allowed to react for 24 h at 70 °C. The protective group was removed using HCl in ethanol, and the product was dried under vacuum. Finally, multi-branched IL chitosan grafted m-PEG was prepared via Schiff base formation reaction between m-PEG-aldehyde and derivatized unprotected chitosan (Scheme 1). To prepare coated magnetic nanoparticles, multi-branched IL chitosan grafted m-PEG was dissolved in water and FeCl_3_·6H_2_O/FeCl_2_·4H_2_O were added to the mixture and stirred at 50 °C for 30 min. Then, ammonia solution was added until the pH of 12, the obtained precipitate separated and washed with water/ethanol and dried under vacuum. The DOX and MTX anticancer drugs’ release performance in both physiological (pH 7.4) and cancer tissue conditions (pH 5.0) was evaluated. At pH 5.0, the cumulative release in the first 48 h was 42.87% and 58.11% with burst release for MTX and DOX, respectively. On the other hand, at pH 7.4, the drugs’ cumulative release was 13.77% for MTX and 22.99% for DOX in the first 48 h. This difference in response to cancer tissue conditions vs. physiological conditions highlights the potential application of this material for cancer treatments.

**Scheme 2:**
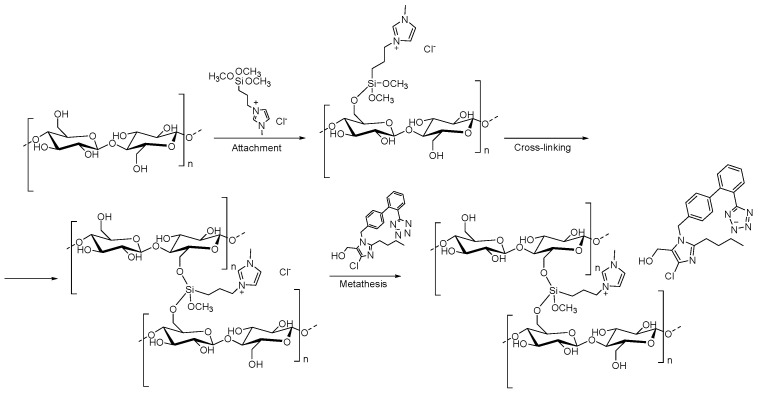
Schematic representation of microcrystalline cellulose grafted with silanized imidazolium ILs as a new controlled release system for Losartan [86].

ILs were also anchored onto a polymeric surface by microcrystalline cellulose grafted with silanized imidazolium ILs for controlled release of Losartan, a medication to treat high blood pressure [86]. Here, pre-synthesized silanized IL 3-methyl-1-[(trimethoxysilyl)propyl]-1H-imidazolium chloride ([(MeO)_3_Si(CH_2_)_3_mim]Cl) was appended onto microcrystalline cellulose (MCC), forming a viscous gel, [MCC—(MeO)_2_Si(CH_2_)_3_mim]Cl (Scheme 2). Because the reaction between MCC and [(MeO)_3_Si(CH_2_)_3_mim]Cl allows for more than just one substitution, cellulose became crosslinked with [(MeO)_3_Si(CH_2_)_3_mim] agent, forming a cross-linked cellulose matrix with charge attracted Cl-anions. The Cl-anions were exchanged for Losartan by metathesis producing [MCC-(MeO)_2_Si(CH_2_)_3_mim][Losartan], Scheme 2.

The Losartan release was conducted in two buffers, with pH = 7.4 (blood pH) and pH = 3.0 (stomach pH). The amount of released Losartan was close at both pH, 88% and 84%, respectively, however, the release at pH = 7.4 was almost 3 times slower than at pH = 3.0 (1085 and 330 min, respectively). Due to the effect of pH on the release rate, this DDS showed promise as a pH-controlled-release system. A patent for a controlled release of drugs from cationic cellulose, produced via functionalization of cellulose with siloxane groups present in IL molecules, expanded the range of ILs and APIs that could be delivered by this method [87]. 

**Scheme 3:**
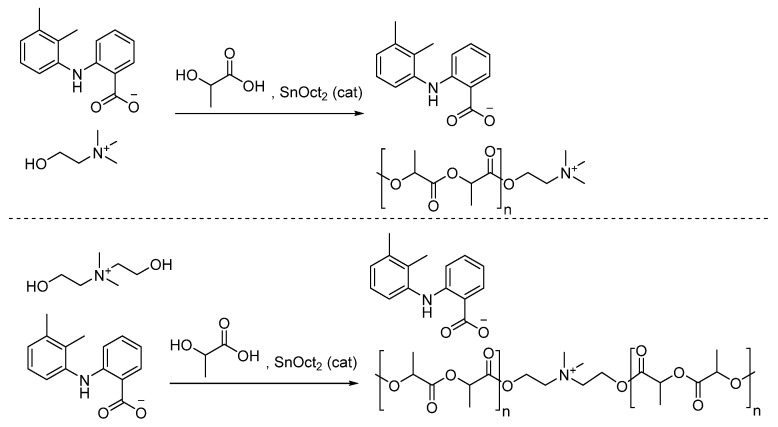
Schematic representation of the preparation of (**top**) poly(L-lactide)-choline and (**bottom**) poly(L-lactide)-di(2-hydroxyethyl)dimethylammonium mefenamates.

While there is no consensus on whether PLA is a biopolymer or rather a bio-based polymer, it is widely used in biomedicine on account of its biocompatibility, biodegradability, and strength. A DDS based on the grafted polylactic acid matrix was reported to deliver mefenamic acid, a nonsteroidal anti-inflammatory drug used to treat mild to moderate pain [88]. For that, two mefenamic acid-based API-ILs, namely, choline mefenamate (Scheme 3, top) and dimethyldihydroxyethylammonium mefenamate (Scheme 3, bottom) were first prepared. After that, the API-ILs reacted with lactic acid monomer in the presence of a catalyst to produce poly(L-lactide)-choline and poly(L-lactide)-di(2-hydroxyethyl)dimethylammonium polymeric matrix, with the mefenamate anion ionically bonded within. Nanoparticles of poly(L-lactide)-choline and poly(L-lactide)-di(2-hydroxyethyl)dimethylammonium mefenamates were then produced using a standard emulsion-solvent evaporation technique, from a water-dichloromethane mixture and PVA that acted as an emulsifier. The nanoparticles exhibited 330 nm (choline-based matrix) or 380 nm (dimethyldiethanolammonium-based matrix) size; the larger size of dimethyldiethanolammonium-based matrix evidently comes from the presence of two reactive hydroxyl groups on the cation and hence larger size of the final molecule. The in vitro drug release from NPs was carried out in saline phosphate buffer (pH 7.4) at body temperature (37 °C). The NPs containing poly(L-lactide)-di(2-hydroxyethyl)dimethylammonium exhibited a drug release profile with two different kinetics profiles, involving a rapid initial burst characterized by a high-speed release followed by sustained release. On the other hand, the NPs of poly(L-lactide)-choline showed sustained release of the drug. In the case of poly(L-lactide)-choline NPs, the drug-release rate was found to be variable and dependent on the amount of drug entrapped in the NPs and the Mw of the polymeric matrix and was slower from the NPs with higher drug loading and larger size.

**Scheme 4:**
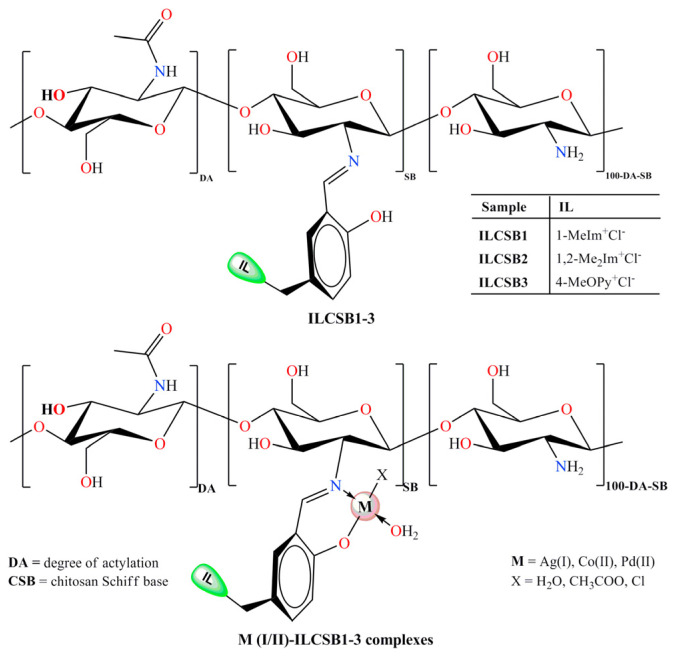
Schematic representation of ILs-anchored chitosan Schiff bases and their metal complexes. Reproduced with permission from [89].

The free amino groups on the surface of chitosan enable a diversity of surface modifications such as acylation, carboxymethylation, cyanoethylation, phosphorylation, and Schiff base condensation. A series of biopolymeric chitosan Schiff bases were generated by condensation of salicylaldehydes ILs (IL-Sal) with chitosan (ILCSB1-3, poly-(GlcNHAc-GlcNH_2_-(GlcN-Sal-IL)), followed by their metalation to produce the corresponding Ag(I)/M(II) complexes (where M = Co, Pd) (Scheme 4) [89]. 

Chitosan anchored Schiff bases and their metal complexes exhibited considerably higher solubility in aqueous media and moderate to excellent broad-spectrum antibacterial efficacy in comparison to the parent chitosan and standard antibiotics, with an ability to inhibit the growth of *Aspergillus flavus* < *Candida albicans* < *Escherichia coli* < *Staphylococcus aureus*. Among assaying compounds, cobalt complexes, [Co(ILCSB2)(OAc)(H_2_O)] and [Co(ILCSB3)(OAc)(H_2_O)], were found to have remarkably potent bactericidal effects (MIC_90_ *S. aureus* = 62 µg/mL; MIC_90_ *C. albicans* = 182 µg/mL) in comparison to standard antibiotic drugs. 

Interestingly, the metal complexes were more potent than their parent ILCSBs and exhibited moderate fungicidal efficacy in relation to the standard antibiotics. In addition, structure–activity relationship for new compounds against human colon carcinoma (HCT-116) cell lines revealed that the antitumor efficacy of metal complexes was superior to that of the parent ligand and their cytotoxic effect was tuned by metal ion and ionic terminal exchange. For example, Ag-ILCSB2 (IC_50_ = 9.13 µg/mL) was ca. 5-fold more cytotoxic against HCT-116 cell lines than ILCSB2 (IC_50_ = 43.30 µg/mL). Furthermore, the Ag(I)-ILCSBs (IC_50_ = 9.13 and 11.1 µg/mL for Ag(I)-ILCSB2 and Ag(I)-ILCSB1, respectively) were more effective in inducing cell death than Co(II)-ILCSB3 (IC_50_ = 24.4 µg/mL) [89]. Further structural refinement and more microbiological assessments may offer new promising antibiotic candidates that play a critical role in fighting staphylococcalcidal and *C. albicans* infections.

## 3. Conclusions

Drug delivery systems (DDSs) allow the drug to be carried throughout the body and protect the drug from degradation while it travels the systemic circulation. While the drug delivery field has advanced drastically in the past few decades, new and improved technologies are constantly being developed. Among DDSs, significant attention is paid to drug delivery vehicles made of biopolymers that allow drugs to travel safely within the human body, have high stability, allow for homogeneous distribution of a drug, and release the drug at an anticipated rate. Polymers can form ultrafine highly porous fiber networks and hence allow for molecular dispersion of the drug within the matrix, limiting its mobility and inhibiting crystal growth and nucleation. Biopolymeric matrices are inert, mechanically strong, and biocompatible. The reviewed literature shows cellulose and chitosan as the most often utilized biopolymers. 

Still, most of the research conducted to date is in the initial stage and should be looked at as proof of concepts of the opportunities that ILs offer. This observation includes the development of solid API-IL which, for example, can exist in polymorphic forms, as it was reported for API-IL ethambutol dibenzoate [90]. There are also cited difficulties with the confirmation of ILs purity due to a lack of analytical protocols. Successful laboratory tests do not necessarily translate into successful clinical trials: while the Lidocainium Etodolac Phase I study was successful, it provided unsatisfactory results in Phases II/III [91]. This being said, many API-ILs are currently in different stages of commercialization [91,92,93].

The drug delivery field is in search of creative solutions and is receptive to new ideas and information arising from different directions and ILs are proposed as an almost infinite toolbox for innovative approaches. This review highlights one more role of ILs in drug delivery, i.e., the use of ILs in the design of DDSs. Although still in its initial stages, this field has demonstrated that:

- ILs can act as solubilizers and stabilizers of drugs;
- Adding ILs into the DDS affects the drug release rate as they participate in both IL-drug and IL-matrix interactions when properly designed. This property of the ILs could be used for slowing drug delivery from biopolymeric matrices;
- Incorporation of the ILs into biopolymeric matrices affects the carrier’s conductivity and its sensitivity to electric stimulation. This anticipates the potential use of these DDSs as stimuli-responsive drug delivery systems for iontophoresis;
- ILs can be used for grafting of the polymers followed by NP coating, to produce a smart magnetic nanocarrier DDS for targeted (multi)drug delivery;
- ILs can be grafted onto polymeric surfaces followed by metathesis, substituting a simple (i.e., Cl^−^) ion with an ionic API. These materials could be employed as adsorption/desorption matrices for ionic APIs;
- ILs can be used for the formation of ILs-anchored chitosan Schiff bases able to form metal complexes with potent bactericidal effects;
- ILs could be employed for the formation of “hybrid gels” containing both the polymer and the ILs, for drug entrapment and release;
- ILs can be used to solubilize the biopolymer and, at the same time, load the drug while the material is formed.

The benefits of using ILs in drug delivery from biopolymeric matrices are evident, and a significant body of information is already accumulated by today. In a world where the presence of plastics and microplastics in our food and water sources and in our bodies is of increasing concern, the use of ILs can facilitate the adoption of DSS based on highly reliable and safe biopolymeric-based DSS. Still, as can be evidenced from the reviewed literature, this field is in its nascent stage, with publications presenting “proof of concepts” of new approaches for more sustainable materials for DDSs. A final application, where the problem is identified, and the performance of these materials is fully demonstrated in the context of that application is, to our opinion, still needed. In addition, identifying the final application, its challenges, and how these new materials can overcome them, will help identify the advantages and disadvantages of one approach over the others.

## References

[1] Earle M.J., Seddon K.R. (2000). Ionic liquids. Green solvents for the future. Pure Appl. Chem..

[2] Wasserscheid K. (2000). Ionic liquids—New “solutions” for transition metal catalysis. Angew. Chem. Int. Ed..

[3] Sirieix J., Ossberger M., Betzemeier B., Knochel P. (2000). Palladium catalyzed cross-couplings of organozincs in ionic liquids. Synlett.

[4] Wasserscheid P., Waffenschmidt H. (2000). Ionic liquids in regioselective platinum-catalysed hydroformylation. J. Mol. Catal. A Chem..

[5] McLachlan F., Mathews C.J., Smith P.J., Welton T. (2003). Palladium-catalyzed Suzuki cross-coupling reactions in ambient temperature ionic liquids: Evidence for the importance of palladium imidazolylidene complexes. Organometallics.

[6] Cole A.C., Jensen J.L., Ntai I., Tran K.L.T., Weaver K.J., Forbes D.C., Davis J.H. (2002). Novel Brønsted acidic ionic liquids and their use as dual solvent−catalysts. J. Am. Chem. Soc..

[7] Schulz P.S., Müller N., Bösmann A., Wasserscheid P. (2007). Effective chirality transfer in ionic liquids through ion-pairing effects. Angew. Chem. Int. Ed..

[8] Yokozeki A., Shiflett M.B. (2009). Separation of Carbon dioxide and sulfur dioxide gases using room-temperature ionic liquid [Hmim][Tf_2_N]. Energy Fuels.

[9] Hough W.L., Smiglak M., Rodríguez H., Swatloski R.P., Spear S.K., Daly D.T., Pernak J., Grisel J.E., Carliss R.D., Soutullo M.D. (2007). The third evolution of ionic liquids: Active pharmaceutical ingredients. New J. Chem..

[10] Rogers R.D., Daly D.T., Swatloski R.P., Hough-Troutman W.L., Troutman J.J.H.L., Marcin S., Juliusz P., Spear S.K. (2007). Multifunctional Ionic Liquid Compositions for Overcoming Polymorphism and Imparting Improved Properties for Active Pharmaceutical, Biological, Nutritional and Energetic Ingredients.

[11] Ferraz R., Branco L.C., Prudêncio C., Noronha J.P., Petrovski Ž. (2011). Ionic liquids as active pharmaceutical ingredients. ChemMedChem.

[12] Uddin M.N., Basak D., Hopefl R., Minofar B. (2020). Potential application of ionic liquids in pharmaceutical dosage forms for small molecule drug and vaccine delivery system. J. Pharm. Pharm. Sci..

[13] Amaral M., Pereiro A.B., Gaspar M.M., Reis C.P. (2021). Recent advances in ionic liquids and nanotechnology for drug delivery. Nanomedicine.

[14] Hough W.L., Rogers R.D. (2007). Ionic liquids then and now: From solvents to materials to active pharmaceutical ingredients. Bull. Chem. Soc. Jpn..

[15] Shamshina J., Kelley S., Gurau G., Rogers R.D. (2015). Chemistry: Develop ionic liquid drugs. Nature.

[16] Bernstein J. (2002). Polymorphism in Molecular Crystals, IUCR Monographs on Crystallography 14.

[17] Schuster D., Laggner C., Langer T. (2005). Why drugs fail—A study on side effects in new chemical entities. Curr. Pharm. Des..

[18] Wojnarowska Z., Paluch M., Grzybowski A., Adrjanowicz K., Grzybowska K., Kaminski K., Wlodarczyk P., Pionteck J. (2009). Study of molecular dynamics of pharmaceutically important protic ionic liquid-verapamil hydrochloride. I. Test of thermodynamic scaling. J. Chem. Phys..

[19] Savjani K.T., Gajjar A.K., Savjani J.K. (2012). Drug solubility: Importance and enhancement techniques. ISRN Pharm..

[20] Morris K.R., Brittain H.G. (1999). Polymorphism in pharmaceutical solids. Drugs and the Pharmaceutical Sciences.

[21] Sarbajna R.M., Preetam A., Sivalakshmi A., Devi A.S., Suryanarayana M.V., Sethi M., Dutta D. (2011). Studies on crystal modifications of Ganciclovir. Mol. Cryst. Liq. Cryst..

[22] Roque-Flores R.L., Guzei I.A., Matos J.D.R., Yu L. (2017). Polymorphs of the antiviral drug ganciclovir. Acta Cryst..

[23] Bauer J., Spanton S., Henry R., Quick J., Dziki W., Porter W., Morris J. (2001). Ritonavir: An extraordinary example of conformational polymorphism. Pharm. Res..

[24] Morissette S.L., Soukasene S., Levinson D., Cima M.J., Almarsson O. (2003). Elucidation of crystal form diversity of the HIV protease inhibitor ritonavir by high-throughput crystallization. Proc. Natl. Acad. Sci. USA.

[25] Frizzo C.P., Gindri I.M., Tier A.Z., Buriol L., Moreira D.N., Martins M.A.P. (2013). Pharmaceutical Salts: Solids to Liquids by Using Ionic Liquid Design. Ionic Liquids: New Aspects for the Future.

[26] Egorova K.S., Gordeev E.G., Ananikov V.P. (2017). Biological activity of ionic liquids and their application in pharmaceutics and medicine. Chem. Rev..

[27] Curreri A.M., Mitragotri S., Tanner E.E.L., Curreri A.M., Mitragotri S., Tanner E.E.L. (2021). Recent advances in ionic liquids in biomedicine. Adv. Sci..

[28] Liu C., Chen B., Shi W., Huang W., Qian H. (2022). Ionic liquids for enhanced drug delivery: Recent progress and prevailing challenges. Mol. Pharmaceutics.

[29] Julio A., Costa Lima S.A., Reis S., Santos de Almeida T., Fonte P. (2020). Development of ionic liquid-polymer nanoparticle hybrid systems for delivery of poorly soluble drugs. J. Drug Delivery Sci. Technol..

[30] Gamboa A., Schüßler N., Soto-Bustamante E., Romero-Hasler P., Meinel L., Morales J.O. (2020). Delivery of ionizable hydrophilic drugs based on pharmaceutical formulation of ion pairs and ionic liquids. Eur. J. Pharm. Biopharm..

[31] Md Moshikur R., Chowdhury M.R., Moniruzzaman M., Goto M. (2020). Biocompatible ionic liquids and their applications in pharmaceutics. Green Chem..

[32] Chowdhury M.R., Moshikur R.M., Wakabayashi R., Tahara Y., Kamiya N., Moniruzzaman M., Goto M. (2019). Development of a novel ionic liquid-curcumin complex to enhance its solubility, stability, and activity. Chem. Commun..

[33] Dobler D., Schmidts T., Klingenhöfer I., Runkel F. (2013). Ionic liquids as ingredients in topical drug delivery systems. Int. J. Pharm..

[34] Li J., Shen L., Yang X., Zhang Y., Liu X., Zhang Y., Xu W. (2021). Novel Acid Response Ionic Liquid Micro Capsule and Its Preparation Method and Application.

[35] Liang P., Yu J., Xu J., Tan L., Ren X., Meng X. (2018). Targeted Microwave Controlled Release Drug-Loaded Nanospheres for Treating Liver Cancer and Preparation Method Thereof.

[36] Cui X., Wang C., Zhang D., Yang X., Xiong Y., Yang Y., Qu Y. (2017). Ionic Liquid Microemulsion and Application Thereof.

[37] Viau L., Tourné-Péteilh C., Devoisselle J.M., Vioux A. (2010). Ionogels as drug delivery system: One-step sol-gel synthesis using imidazolium ibuprofenate ionic liquid. Chem. Commun..

[38] Nor S.B.M., Woi P.M., Ng S.H. (2017). Characterisation of ionic liquids nanoemulsion loaded with piroxicam for drug delivery system. J. Mol. Liq..

[39] Ziółkowski B., Diamond D. (2013). Thermoresponsive poly(ionic liquid) hydrogels. Chem. Commun..

[40] Cardinal J.R., Anderson J.M., Kim S.W. (1984). Drug Release from Matrix Devices. Recent Advances in Drug Delivery Systems.

[41] Bica K., Rodriguez H., Gurau G., Cojocaru O.A., Riisager A., Fehrmann R., Rogers R.D. (2012). Pharmaceutically active ionic liquids with solids handling, enhanced thermal stability, and fast release. Chem. Commun..

[42] Boyd B.J., Bergström C.A.S., Vinarov Z., Kuentz M., Brouwers J., Augustijns P., Brandl M., Bernkop-Schnürch A., Shrestha N., Préat V. (2019). Successful oral delivery of poorly water-soluble drugs both depends on the intraluminal behavior of drugs and of appropriate advanced drug delivery systems. Eur. J. Pharm. Sci..

[43] Klemm D., De Baets S., Vandamme E., Steinbüchel A.T. (2004). Cellulose. Biopolymers in 10 Volumes.

[44] Barikani M., Oliaei E., Seddiqi H., Honarkar H. (2014). Preparation and application of chitin and its derivatives: A review. Iran. Polym. J..

[45] García M.C., Parambath A. (2018). Drug delivery systems based on nonimmunogenic biopolymers. Engineering of Biomaterials for Drug Delivery Systems.

[46] Courtenay J.C., Sharma R.I., Scott J.L. (2018). Recent advances in modified cellulose for tissue culture applications. Molecules.

[47] Hickey R.J., Pelling A.E. (2019). Cellulose biomaterials for tissue engineering. Front. Bioeng. Biotech..

[48] Song J.H., Murphy R.J., Narayan R., Davies G.B.H. (2019). Biodegradable and compostable alternatives to conventional plastics. Philos. Trans. R. Soc. Lond. B.

[49] Clark W.R., Gao D. (2002). Properties of membranes used for hemodialysis therapy. Semin. Dial..

[50] Deppisch R., Storr M., Buck R., Göhl H. (1998). Blood material interactions at the surfaces of membranes in medical applications. Sep. Purif. Technol..

[51] Xu Z.-K., Huang X.-J., Wan L.-S. (2009). 7: Membranes with Glycosylated Surface. Surface Engineering of Polymer Membranes. Advanced Topics in Science and Technology in China.

[52] Sindhu K.A., Prasanth P., Thakur V.K. (2014). Medical Applications of Cellulose and Its Derivatives: Present and Future. Nanocellulose Polymer Nanocomposites: Fundamentals and Applications.

[53] Foster A.B., Webber J.M., Melville L., Wolfrom R., Tipson S. (1961). Chitin. Advances in Carbohydrate Chemistry.

[54] Mi F.-L., Shyu S.-S., Lin Y.-M., Wu Y.-B., Peng C.-K., Tsai Y.-H. (2003). Chitin/PLGA blend microspheres as a biodegradable drug delivery system: A new delivery system for protein. Biomaterials.

[55] Wan A.C.A., Tai B.C.U. (2013). Chitin—A promising biomaterial for tissue engineering and stem cell technology. Biotech. Adv..

[56] Funakoshi T., Majima T., Suenaga N., Iwasaki N., Yamane S., Minami A. (2006). Rotator cuff regeneration using chitin fabric as an acellular matrix. J. Shoulder Elb. Surg..

[57] Swatloski R.P., Spear S.K., Holbrey J.D., Rogers R.D. (2002). Dissolution of cellulose with ionic liquids. J. Am. Chem. Soc..

[58] Swatloski R.P., Holbrey J.D., Rogers R.D. (2003). Dissolution and Processing of Cellulose Using Ionic Liquids.

[59] Remsing R.C., Swatloski R.P., Rogers R.D., Moyna G. (2006). Mechanism of cellulose dissolution in the ionic liquid1-n-butyl-3-methylimidazolium chloride: A 13C and 35/37Cl NMR relaxation study on model systems. Chem. Commun..

[60] Rabideau B.D., Ismail A.E. (2015). Mechanisms of hydrogen bond formation between ionic liquids and cellulose and the influence of water content. Phys. Chem. Chem. Phys..

[61] Kostag M., Jedvert K., Achtel C., Heinze T., El-Seoud O.A. (2018). Recent advances in solvents for the dissolution, shaping and derivatization of cellulose: Quaternary ammonium electrolytes and their solutions in water and molecular solvents. Molecules.

[62] Rahman M., Rodriguez H., Sun N., Swatloski R.P., Daly D.T., Rogers R.D. (2014). Ionic Liquid Systems for the Processing of Biomass, Their Components and/or Derivatives, and Mixture Thereof. U.S. Patent.

[63] Qin Y., Lu X., Sun N., Rogers R.D. (2010). Dissolution or extraction of crustacean shells using ionic liquids to obtain high molecular weight purified chitin and direct production of chitin films and fibers. Green Chem..

[64] Wang H., Gurau G., Rogers R.D., Zhang S., Wang J., Lu X., Zhou Q. (2014). Dissolution of Biomass Using Ionic Liquids. Structures and Interactions of Ionic Liquids.

[65] Wu Y., Sasaki T., Irie S., Sakurai K. (2008). A novel biomass-ionic liquid platform for the utilization of native chitin. Polymer.

[66] Turner M.B., Spear S.K., Holbrey J.D., Daly D.T., Rogers R.D. (2005). Ionic liquid-reconstituted cellulose composites as solid support matrices for biocatalyst immobilization. Biomacromolecules.

[67] Turner M.B., Spear S.K., Holbrey J.D., Rogers R.D. (2004). Production of bioactive cellulose films reconstituted from ionic liquids. Biomacromolecules.

[68] Shamshina J.L., Rogers R.D. (2018). Chitin nanobeads. Biopolymeric microbeads as alternatives to synthetic plastics. H PC Today.

[69] Shamshina J.L., Zavgorodnya O., Choudhary H., Frye B., Newbury N., Rogers R.D. (2018). In search of stronger/cheaper chitin nanofibers through electrospinning of chitin–cellulose composites using an ionic liquid platform. ACS Sustain. Chem. Eng..

[70] Shamshina J.L., Zavgorodnya O., Bonner J.R., Gurau G., DiNardo T., Rogers R.D. (2016). “Practical” electrospinning of biopolymers in ionic liquids. ChemSusChem.

[71] Shamshina J.L., Abidi N. (2021). Choosing the right strategy: Cryogrinding vs. ball milling—Comparing apples to apples. Green Chem..

[72] Shamshina J.L., Gurau G., Block L.E., Hansen L., Dingee C., Walters A., Rogers R.D. (2014). Chitin–calcium alginate composite fibers for wound care dressings spun from ionic liquid solution. J. Mater. Chem. B.

[73] Shamshina J.L., Zavgorodnya O., Berton P., Chhotaray P.K., Choudhary H., Rogers R.D. (2018). Ionic liquid platform for spinning composite chitin-poly(lactic acid) fibers. ACS Sustain. Chem. Eng..

[74] Oprea M., Voicu S.I. (2020). Cellulose composites with graphene for tissue engineering applications. Materials.

[75] Hai T.A.P., Sugimoto R. (2018). Fluorescence control of chitin and chitosan fabricated *via* surface functionalization using direct oxidative polymerization. RSC Adv..

[76] Morais E.S., Silva N.H.C.S., Sintra T.E., Santos S.A.O., Neves B.M., Almeida I.F., Costa P.C., Correia-Sá I., Ventura S.P.M., Silvestre A.J.D. (2019). Anti-inflammatory and antioxidant nanostructured cellulose membranes loaded with phenolic-based ionic liquids for cutaneous application. Carbohydr. Polym..

[77] Chantereau G., Sharma M., Abednejad A., Neves B.M., Sèbe V.C., Freire M.G., Freire C.S.R., Silvestre A.J.D. (2019). Design of nonsteroidal anti-inflammatory drug-based ionic liquids with improved water solubility and drug delivery. ACS Sustain. Chem. Eng..

[78] Sastry N.V., Singh D.K., Trivedi P.A. (2018). Hybrid hydrogel systems of micelles of drug anion containing ionic liquid and biopolymers: Rheological behavior and drug release. Colloids Surf. A.

[79] Zhang X., Wang W., Zhang J., Feng H., Yan J. (2021). Biological-Friendly Slow-Release Nanofiber and Preparation Method Thereof (Mechanical Translation).

[80] Viswanathan G., Murugesan S., Pushparaj V., Nalamasu O., Ajayan P.M., Linhardt R.J. (2006). Preparation of biopolymer fibers by electrospinning from room temperature ionic liquids. Biomacromolecules.

[81] Park T.-J., Lee S.-H., Simmons T.J., Martin J.G., Mousa S.A., Snezhkova E.A., Sarnatskaya V.V., Nikolaev V.G., Linhardt R.J. (2008). Heparin–cellulose–charcoal composites for drug detoxification prepared using room temperature ionic liquids. Chem. Commun..

[82] Hou L., Udangawa W.M.R.N., Pochiraju A., Dong W., Zheng Y., Linhardt R.J., Simmons T.J. (2016). Synthesis of heparin-immobilized, magnetically addressable cellulose nanofibers for biomedical applications. ACS Biomater. Sci. Eng..

[83] Dias A.M.A., Cortez A.R., Barsan M.M., Santos J.B., Brett C.M.A., de Sousa H.C. (2013). Development of greener multi-responsive chitosan biomaterials doped with biocompatible ammonium ionic liquids. ACS Sustain. Chem. Eng..

[84] Safdar R., Gnanasundaram N., Appusamy A., Thanabalan M. (2021). Synthesis, physiochemical properties, colloidal stability evaluation and potential of ionic liquid modified CS-TPP MPs in controlling the release rate of insulin. J. Drug Delivery Sci. Tech..

[85] Rahimi M., Shafiei-Irannejad V., Safa K.D., Salehi R. (2018). Multi-branched ionic liquid-chitosan as a smart and biocompatible nano-vehicle for combination chemotherapy with stealth and targeted properties. Carbohydr. Polym..

[86] Santos S.S.O., Millan R.D.S., Speziali M.G. (2020). Versatile grafted microcrystalline cellulose with ionic liquid as new Losartan-controlled release material. Eur. Polym. J..

[87] Santos S.S.O., Millan R.D.S., Speziali M.G. (2020). Process, for Anchoring Ionic Liquid in Cellulose, Cellulose-Based Products and Use as Adsorption/Desorption Matrix for Anionic Components.

[88] Halayqa M., Zawadzki M., Domańska U., Plichta A. (2017). API-ammonium ionic liquid—Polymer compounds as a potential tool for delivery systems. J. Molec. Liq..

[89] Elshaarawya R.F.M., Refaeec A.A., El-Sawi E.A. (2016). Pharmacological performance of novel poly-(ionic liquid)-grafted chitosan-N-salicylidene Schiff bases and their complexes. Carbohydr. Polym..

[90] Cherukuvada S., Nangia A. (2012). Polymorphism in an API ionic liquid: Ethambutol dibenzoate trimorphs. CrystEngComm.

[91] Lu Y., Qi J., Wu W. (2022). Ionic liquids-based drug delivery: A perspective. Pharm. Res..

[92] Licart. https://www.licart.com/.

[93] Centene Corporation Clinical Policy: Diclofenac (Cambia, Flector, Licart, Pennsaid, Solaraze, Zipsor, Zorvolex). https://www.superiorhealthplan.com/content/dam/centene/Superior/policies/pharmacy-policies/CP.PCH.28-12012021.pdf.

